# Non-Hodgkin Lymphoma in the Middle East Is Characterized by Low Incidence Rates With Advancing Age

**DOI:** 10.1200/JGO.18.00241

**Published:** 2019-04-12

**Authors:** Rafil T. Yaqo, Sana D. Jalal, Kharaman J. Ghafour, Hemin A. Hassan, Michael D. Hughson

**Affiliations:** ^1^ Duhuk University School of Medicine, Dohuk, Iraq; ^2^ Sulaimania University College of Medicine, Sulaimaniyah, Iraq; ^3^ Shorsh General and Hiwa Cancer Hospitals, Sulaimaniyah, Iraq

## Abstract

**PURPOSE:**

In the Middle East, incidence rate ratios (IRRs) of non-Hodgkin lymphoma (NHL) to Hodgkin lymphoma (HL) are more than 50% lower than the United States.

**MATERIALS AND METHODS:**

Age-specific incidence rates (ASIRs), age-adjusted incidence rates (AAIRs), and IRRs of NHL:HL were compared using the cancer registries of Iraq, Jordan, Saudi Arabia, and US SEER.

**RESULTS:**

The NHL AAIR (95% CI) per 100,000 population was 4.4 (4.1 to 4.7) for Iraq, 5.4 (4.6 to 6.2) for Jordan, 4.7 (4.4 to 5.1) for Saudi Arabia, and 13.2 (13.0 to 13.4) for the United States. The HL AAIR was 1.8 (1.6 to 2.0) for Iraq, 1.8 (1.4 to 2.2) for Jordan, 2.1 (1.9 to 2.2) for Saudi Arabia, and 2.3 (2.2 to 2.4) for the United States, with respective NHL:HL IRR of 2.4 (2.2 to 2.7), 3.0 (2.4 to 3.8), 2.2 (2.0 to 2.5), and 5.7 (5.5 to 6.0). NHL ASIRs for the Middle East and the United States were similar until 30 to 39 years of age. Thereafter, ASIR of NHL peaked at 20 to 33 per 100,000 at age 70 years in the Middle East regions, all much lower than the US age 70 years rate of greater than 100 per 100,000. Diffuse large B-cell lymphoma (DLBCL) represented 52% of NHL in Sulaimaniyah Province of Iraq and 51% of NHL in Saudi Arabia. Both regions had AAIR for DLBCL less than 42% of DLBCL in US SEER. Pediatric Epstein-Barr virus–related Burkitt’s lymphoma at 8% was the second most frequent NHL in Sulaimaniyah but made little contribution to overall NHL rates.

**CONCLUSION:**

The incidence of HL was slightly lower than in the United States, but it was the markedly lower rates of adult NHL with advancing age, including the predominant DLBCL, that accounted for the low NHL:HL IRR in these Middle Eastern countries.

## INTRODUCTION

In most of the Middle East, the relative frequency of non-Hodgkin lymphoma (NHL) seems to be low compared with Hodgkin lymphoma (HL). This is commonly measured as incidence rate ratio (IRR) with non–age-adjusted IRR of NHL:HL from the National Cancer Registries of Iraq and Jordan reported as 2.0 and 1.8, respectively.^1,2^ This contrasts with the worldwide age-adjusted IRR of 5.2 in Globocan and 9.0 in the Global Burden of Disease.^3,4^

The Kurdish Province of Sulaimaniyah has a well-defined population, with public cancer services that provide a comprehensive registration of patients and centrally reviewed diagnoses.^5,6^ Jordan and Saudi Arabia have population-based cancer registries that meet criteria for submitting data to the International Association for Research on Cancer (IARC).^7^ The Cancer Registry of Iraq is not a part of the IARC but collects provincial data and has recently published national statistics that are similar to other large Middle Eastern countries and seem to accurately reflect cancer risk in the country.^1^

In this study, estimates of age-specific incidence rates (ASIRs) and age-adjusted incidence rates (AAIRs) of NHL and HL are compared between Iraq, Jordan, Saudi Arabia, and the US SEER program. The goal was to investigate whether there is a common epidemiologic pattern of lymphoma in this central region of the Middle East that might account for what seems to be the aberrantly low IRR of NHL:HL. In Sulaimaniyah Province, the incidence of diffuse large B-cell lymphoma (DLBCL) and Burkitt’s lymphoma (BL), the most common NHL in our region, was calculated to estimate their contribution to overall lymphoma rates.

## MATERIALS AND METHODS

In 2005, Hiwa Cancer Hospital was established for public cancer care and to provide a population-based cancer registry. The Shorsh Hospital Pathology Department provides central pathology review for Hiwa Hospital patients. Data were obtained from Hiwa Hospital and Shorsh Pathology Department for patients registered between January 1, 2010 and December 31, 2014 as residents of Sulaimaniyah Province. Iraq data were obtained from the 2010 Iraqi Cancer Registry,^1^ Jordanian data from the 2012 Jordan Cancer Registry,^2^ Saudi Arabian data from the 2014 Saudi Arabia Cancer Registry,^8^ and US data from the SEER Cancer Statistic Review, 1975 to 2014.^9^

### Calculation of ASIR and AAIR

Lymphomas for all regions were sorted into the 10-year age ranges 0 to 9, 10 to 19, 20 to 29, 30 to 39, 40 to 49, 50 to 59, 60 to 69, and 70 years or older. The 70 years and older age range was the upper limit used in the Iraqi Cancer Registry and, for uniformity, was applied to all regions. For Sulaimaniyah, incidence was calculated on the average number of cases in the 10-year age ranges for the 5-year period 2010 to 2014. For Iraq (2010), Jordan (2014), and Saudi Arabia (2014), the cancer registries provided the number of NHLs and HLs for the single indicated year. For US SEER, ASIRs were published in 5-year age ranges.^9^ From the 5-year ASIR, the number of tumors in 10-year intervals and 10-year ASIRs were estimated by extrapolating to 100 million persons using the US 2010 population distribution.^10^

For all regions, ASIRs were calculated based on the number of lymphomas per 100,000 persons in the specified 10-year age groups. AAIRs were calculated from ASIRs adjusted by the 2002 WHO World Standard Population.

### Analysis of Sulaimaniyah Lymphoma Subtypes

Sections were prepared for standard hematoxylin and eosin stains and for immunohistochemical staining as previously reported.^11^ Most BL and low-grade lymphomas were immunophenotyped by flow cytometry at the Sulaimaniyah Public Health Laboratory (S.D.J.). On the basis of morphology, immunohistochemistry, and flow cytometry, cases were diagnosed as HL or a specific category of NHL according to the WHO 2008 classification of tumors of lymphoid tissue.^11^

### Epstein-Barr Virus in HL and Epstein-Barr Virus and MYC Translocation in BL

Epstein-Barr virus (EBV) latency was assessed by in situ hybridization using the probes (Novocastra; Leica Biosystems, Wetzler, Germany for BL and ZytoFast EBV-CISH system; ZytoVision, Bremerhaven, Germany for HL) against EBV-encoded small RNAs (EBER1 and EBER2). *C-MYC/IGH* translocations were analyzed by fluorescence in situ hybridization using a break-apart probe (Vysis *MYC*; Abbott, Abbott Park, IL). The studies used formalin-fixed, paraffin-embedded tissue on 22 BLs and 33 HLs.

### Data Analysis

Data were entered into Excel worksheets and analyzed with Excel mathematical functions or Stata IC10 (STATA, College Station, TX) statistical software. Uncertainty was analyzed by comparing 95% CIs for ASIR, AAIR, and IRR in the different geographic regions. The calculation of CI was based on the following formulas for directly age-adjusted rates^12^ and rate ratios^13^:

$$95\%\;CI\left(ASIR\right)=1.96\times \left(\sqrt{Ri^{2}/Ni}\right)$$

where Ri = age-specific incidence in the 10-year age group, and Ni = number of tumors in the age-specific age group.

95% CI(AAIR)=1.96×SE(standard error)AAIR where

$$AAIR\;var\left(variance\right)=\sum Wsi^{2}\times Ri^{2}/Niand$$

$$SE\left(AAIR\right)=\sqrt{AAIRvar}$$

where Wsi = weight in the standard population of the 10-year age group.

95% CI (IRR) = antilog (e) of lower and upper bounds of the natural log of the IRR,

$${\text{e}}^{\text{logIRR}\pm \left[\text{1}\text{.96}\times \sqrt{\text{1}/\text{N1}+1/N2}\right]}$$

$$Lower\;bound=logIRR-\left[\text{1}\text{.96}\times \sqrt{\text{1}/\text{N1}+1/N2}\right]$$

$$Upper\;bound=logIRR+\left[\text{1}\text{.96}\times \sqrt{\text{1}/\text{N1}+1/N2}\right]$$

where N1 = number of lymphomas for population 1, and N2 = number of lymphomas for population 2.

Differences were considered significant if the 95% CI did not overlap. For HL, a ratio of ASIRs and AAIRs for Iraq, Jordan, and the United States were indexed to Saudi Arabia. The differences in IRR were considered significantly different than Saudi Arabia if the 95% CI did not include 1.

## RESULTS

### Lymphoma Subtypes

In the 5-year period 2010 to 2014, 128 cases of HL and 406 cases of NHL were diagnosed in the Sulaimaniyah Province, with 88% being B cell and 12% T cell (Table 1). DLBCL diagnosed at a median age of 54 years was the most common subtype at 52% of all NHLs. BL diagnosed at a median age of 5 years was the second most frequent at 8%. HL was predominantly nodular sclerosis (73%), with mixed cellularity representing 15% of cases and with no significant difference in age between the two subtypes (*P* = .95). Lymphocyte-rich and lymphocyte-depleted classic HL was diagnosed in 9% of cases. Five nodular lymphocyte–predominant HLs (3%) were reported in the 5-year period.

**TABLE 1:**
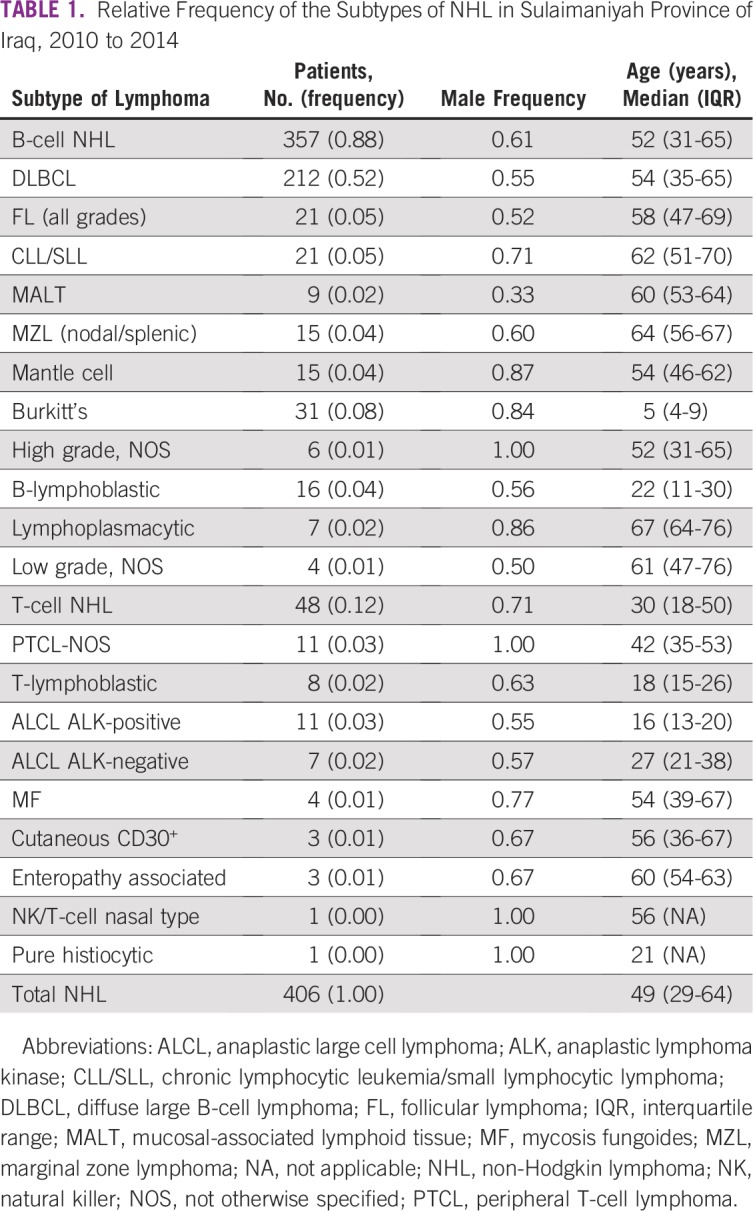
Relative Frequency of the Subtypes of NHL in Sulaimaniyah Province of Iraq, 2010 to 2014

For all Iraq, 953 cases of NHL are designated as histologically verified, but the subtyping did not follow WHO guidelines, and diagnoses were listed as: lymphoma, non-Hodgkin, 561 (59%); lymphoma, 343 (36%); lymphoma large-cell diffuse, 16 (2%); and others including BL and follicular lymphoma at 1% or less each.^1^ The Jordan cancer registry did not provide any lymphoma subtyping.^2^ In Saudi Arabia, 51% of NHL was reported as diffuse large B-cell lymphoma, 7% as follicular lymphoma, and 4% as BL.^8^ For Saudi Arabia HL, 58% was nodular sclerosis and 11% nodular lymphocyte–predominant HL.

### Age-Specific and Age-Standardized Incidence Rates of HL and NHL

Table 2 provides the distribution of the total populations and the number of NHLs and HLs in 10-year intervals for Sulaimaniyah, Iraq, Jordan, Saudi Arabia, and US SEER. All of the Middle East regions have a young age structure, with 75% to 82% of the populations being younger than 40 years old.

**TABLE 2:**
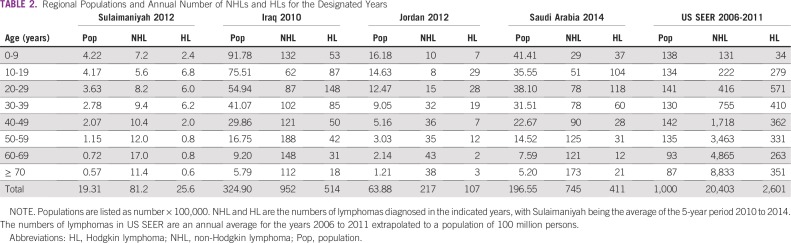
Regional Populations and Annual Number of NHLs and HLs for the Designated Years

Male to female IRRs were nearly the same for both NHL and HL in each region except HL in Jordan, where more females were recorded than males (Table 3). This was a seemingly aberrant 1-year occurrence; previous multiyear Jordanian studies have shown a 1.4:1 male to female HL ratio.^14^ We do not believe that there are any significant regional sex differences, and subsequent analyses were for total populations. The ASIRs of each region are shown for NHL (Table 4) and HL (Table 5) together with the AAIR and the 95% CI for each calculation.

**TABLE 3:**
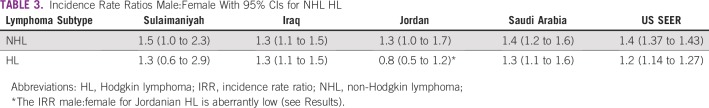
Incidence Rate Ratios Male:Female With 95% CIs for NHL HL

**TABLE 4:**
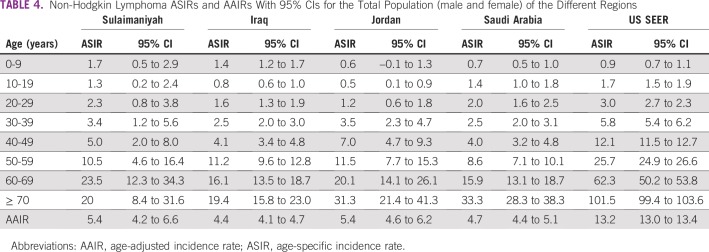
Non-Hodgkin Lymphoma ASIRs and AAIRs With 95% CIs for the Total Population (male and female) of the Different Regions

**TABLE 5:**
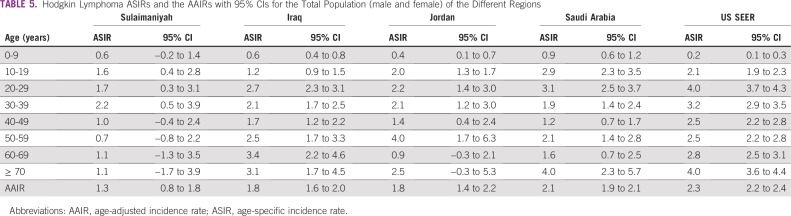
Hodgkin Lymphoma ASIRs and the AAIRs with 95% CIs for the Total Population (male and female) of the Different Regions

**FIG 1:**
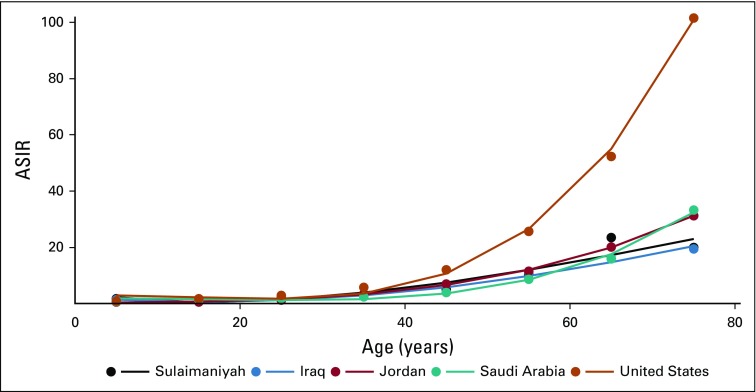
Non-Hodgkin lymphoma. Changes in age-specific incidence rate (ASIR) with increasing age. ASIR is similar in all regions until approximately age 30 years, when US rates begin to increase. At 70 years of age and older, US rates are nearly three times those of the Middle East, where the small differences are not significant. Black, Sulaimaniyah; blue, Iraq; purple, Jordan; teal, Saudi Arabia; dark orange, United States.

For NHL, the 95% CIs of the AAIRs for each of the Middle East regions overlapped with each other, as did the 95% CIs of the ASIRs for each of the 10-year intervals (except for the 70 years or older group between Iraq and Saudi Arabia). We interpret these findings as indicating that there are no significant differences in the 10-year ASIR or the AAIR between the Middle Eastern regions for NHL. The NHL 10-year age-specific rates for the United States began to diverge from those of the Middle East at approximately 30 years of age, by age 60 years doubled, and after age 70 years more than tripled Middle Eastern rates (Fig 1).

For HL, the AAIRs were similar for Iraq, Jordan, and Saudi Arabia. In all of the Middle East registries, most HL was found in those younger than 40 years of age (73% in Iraq; 78% in Jordan and Saudi Arabia), whereas in US SEER, 50% of HL was diagnosed after 39 years of age. Jordan and the country of Iraq had attenuated late peaks compared with the United States, and Saudi Arabia had a late peak that matched the United States at 70 years of age or older.

**FIG 2:**
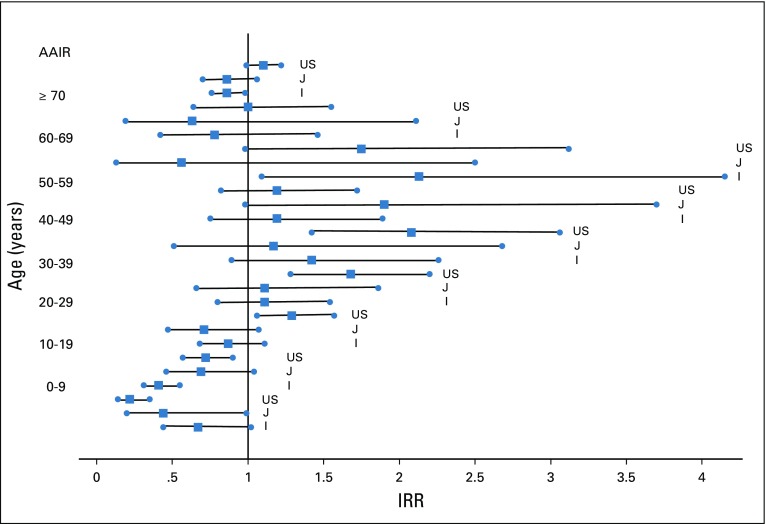
Incidence rate ratios (IRRs) of Iraq (I), Jordan (J), and US Hodgkin lymphoma age-specific and age-adjusted incidence rates (AAIRs) indexed to Saudi Arabia. Saudi Arabia is represented by an incidence rate = 1. The solid squares represent the IRR and the bars the 95% CIs for the IRR. Age-specific incidence rates are indicated by the age ranges. AAIRs are represented by the top three IRRs.

HL rates for Sulaimaniyah were low and changed little after 40 years of age, but the low numbers and the wide 95% CI precluded any regional comparisons. Regional comparisons, therefore, used only national data indexed as an IRR to Saudi Arabia. Saudi Arabia was chosen as the index, because it had the IARC-affiliated registry with the largest number of cases. These comparisons are shown as a Forest plot in Figure 2 (Data Supplement). The HL IRR of the United States was markedly lower than Saudi Arabia at 0 to 9 years of age but was significantly higher from 20 to 50 years of age. The indexed AAIR showed no significant differences in total rates of HL between Saudi Arabia and Iraq, Jordan, or the United States.

### Comparison of NHL:HL IRRs

The NHL:HL IRR (95% CI) for Iraq at 2.4 (2.2 to 2.7), Jordan at 3.0 (2.4 to 3.8), and Saudi Arabia at 2.2 (2.0 to 2.5) were similar to each other and significantly lower than the US rate at 5.7 (5.5 to 6.0; Table 6). The Sulaimaniyah rate of 4.2 (2.7 to 6.5) was not significantly different from the United States at 5.7 (5.5 to 6.0) or the other Middle East regions, with this overlap reflecting the small Sulaimaniyah HL sample size and its wide 95% CI.

**TABLE 6:**
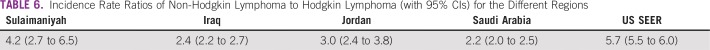
Incidence Rate Ratios of Non-Hodgkin Lymphoma to Hodgkin Lymphoma (with 95% CIs) for the Different Regions

### DLBCL in Sulaimaniyah and Estimates for Saudi Arabia

DLBC represented 52% of NHL in Sulaimaniyah, with an AAIR of 2.9 per 100,000. DLBCL represented 51% of NHL in Saudi Arabia. If it is assumed that these DLBCLs had the same age distribution as Sulaimaniyah, the Saudi Arabia AAIR was 2.6 per 100,000. In the United States, the AAIR for DLBCL was 6.9 per 100,000, a factor 2.4 times greater than Sulaimaniyah or Saudi Arabia. The ASIR for DLBCL in Sulaimaniyah peaked at 13.3 per 100,000 persons at 60 to 69 years old, whereas in the United States, the ASIR of DLBCL was 33.5 per 100,000 after 65 years of age.^9^

### BL Sulaimaniyah

NHLs in Sulaimaniyah, with childhood defined as age 19 years or younger, are presented in Table 7. BL represented 75% of all childhood NHL in those younger than 10 years of age, with 68% being intestinal. The annual incidence of BL was 16.7 per million children for males and 2.5 per million for females.

**TABLE 7:**
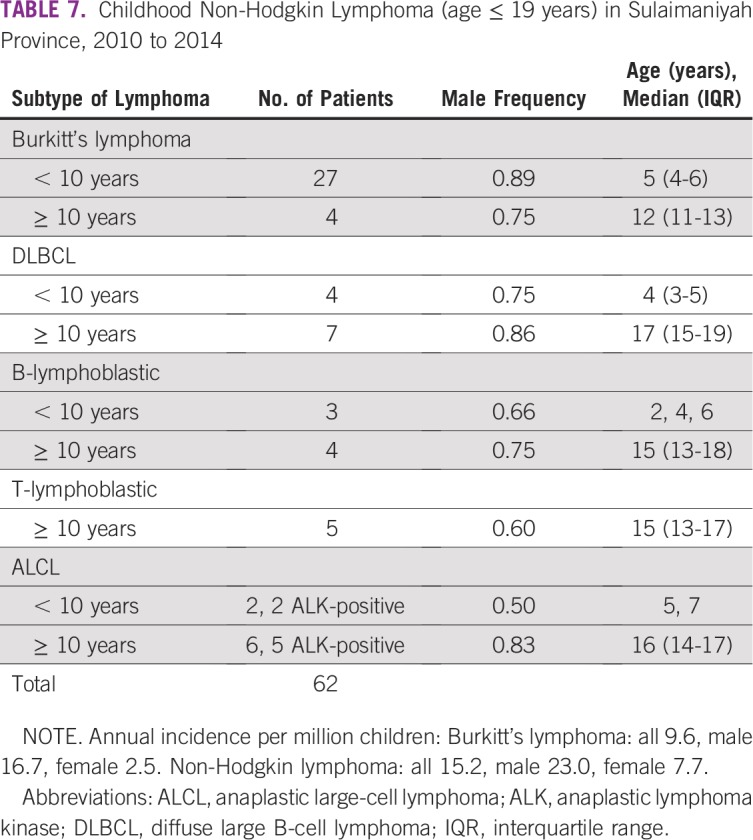
Childhood Non-Hodgkin Lymphoma (age ≤ 19 years) in Sulaimaniyah Province, 2010 to 2014

### EBV EBER and C-MCY Translocations in BL and EBV EBER in HL

All BLs were positive for C-MYC/IGH translocations, and 20 of the 22 cases showed positive nuclear staining for EBER. The two negative EBER cases were not tested for RNA integrity. Four of nine mixed-cellularity HLs (44%), six of 24 nodular sclerosis (25%), and a total of 30% of HLs were EBER positive. The difference in the HL subtype proportions was not significant (Fisher exact test, *P* = .23), nor was the age difference of patients with HL by EBV status significant (EBV-positive, 28.1 ± 21.1 years; EBV-negative, 36.0 ± 19.9 years; *P* = .31).

## DISCUSSION

In this study, the IRRs (95% CI) of NHL:HL in Iraq of 2.4 (2.2 to 2.7), in Jordan of 3.0 (2.4 to 3.8), and in Saudi Arabia of 2.2 (2.0 to 2.5) were similar to each other but significantly lower than the United States at 5.7 (5.5 to 6.0).^3,4,9^ It is further shown that the low Middle Eastern IRRs were the result of a relative infrequency of NHL in late middle age and the elderly. Until early adulthood, the ASIRs of NHL from the Middle East and the United States were nearly identical, and it was just before 40 years of age that US NHL rates began to increase above and after age 70 years to nearly triple Middle Eastern rates.^9^

The lower incidence of NHL in the Middle East can be partly attributed to the low frequency of follicular lymphoma.^15,16^ Nevertheless, an AAIR less than 42% of the US rate was estimated for DLBCL in Sulaimaniyah and Saudi Arabia, which at diagnostic frequencies of 52% and 51% were by far the most common NHL in these regions, with a 56% frequency of DLBCL being reported from Jordan.^17^ In the United States, a 2.3 times higher AAIR of DLBCL compared with the Middle East comes at a lower diagnostic frequency of 22% among US SEER NHL, emphasizing that diagnostic frequency and disease incidence are unrelated.^9^

The indexing of HL rates to Saudi Arabia showed no significant difference in AAIR (95% CI) between Saudi Arabia (1.0), Iraq at 0.9 (0.8 to 1.0), Jordan at 0.9 (0.7 to 1.1), or the United States at 1.1 (1.0 to 1.2). All of the Middle East regions had late age peaks, but more than 70% to 75% of the HL was found before 40 years of age, whereas in the United States, 50% of HL was diagnosed after 39 years of age. It seems notable that the slightly higher rate of HL in the United States compared with the Middle East was not the result of late age peaks but rather of a generally increased rate of HL from 30 to 60 years of age. It is also notable that until 10 years of age, the incidence of HL was markedly lower in the United States compared with Iraq or Saudi Arabia, a likely reflection of the known low rates of HL in young children of the United States.^18^

Childhood rates for NHL were similar to that reported from US SEER, with the exception of BL. This BL is common in many Middle Eastern countries^17-20^ but may be half as frequent in Saudi Arabia at 4% of NHL as in Sulaimaniyah and Jordan at 8%.^8,17^ It has a marked male predominance, usually arises in the terminal ileum, and contains EBV genomic material in nearly all tumors.^19-21^ The estimated incidence of BL at 16.7 per million male children in Sulaimaniyah was more than four times greater than the 3.8 per million males reported for US patients in the pediatric registry of the IARC but considerably less than rates of endemic BL from Uganda at 30.0/million males.^18^ Whether the BL of the Middle East is sporadic or endemic is uncertain and probably lies somewhere in between.^19^

If high rates of BL are related to EBV exposure in young children, this is a curiosity, because early EBV exposure in underdeveloped countries has been associated with childhood mixed-cellularity HL, whereas the later exposure seen in developed countries is associated with adolescent and adult nodular sclerosis.^21,22^ In Iraq, there has been a shift over the past decades from a predominance of mixed-cellularity to nodular sclerosing HL.^11,23^ This may indicate a change in EBV exposure that is affecting HL but not BL.

Nodular sclerosis represents 58% of HL in Saudi Arabia and Jordan and more than 70% of HL in Northern Iraq.^8,11,23,24^ We found that 30% of Iraqi HLs expressed EBV RNA, a proportion similar to that of non–HIV-infected patients in the United States.^25^ Nodular lymphocyte–predominant HL that contains EBV RNA in only 3% to 5% of cases^26^ is reported as 7% to 11% of HL in Saudi Arabia and Jordan. In the United States, nodular lymphocyte–predominant HL comprises approximately 10% and nodular sclerosis 70% of HL, making the Middle Eastern and US patterns of HL subtypes and EBV expression comparable.^25^

The low rates of NHL in the elderly of Middle East may seem peculiar but are also seen with other cancers.^5,6^ The similarity of the NHL rates in Iraq, Jordan, and Saudi Arabia indicates a region-wide phenomenon, and it is unlikely that undue numbers of lymphomas are being undiagnosed at any age.

We suggest that an age cohort effect may be occurring in which high noncancer deaths in older members of Middle East populations reduce their risk of NHL.^9,27^ Life expectancy and the proportion of persons older than 64 years is 69.8 years and 1.8% in Iraq, 74.3 years and 3.3% in Jordan, 74.8 years and 4.1% in Saudi Arabia, and 78.5 years and 14.1% in the United States (Table 8).^1,2,8,27,28^ The Middle East elderly have high age-specific coronary artery disease death rates and correspondingly low cancer incidence rates (Data Supplement).^27^ This implies that coronary artery disease deaths beginning at approximately 45 to 55 years of age may diminish the numbers of older survivors who might develop lymphoma.^27,29^

**TABLE 8:**
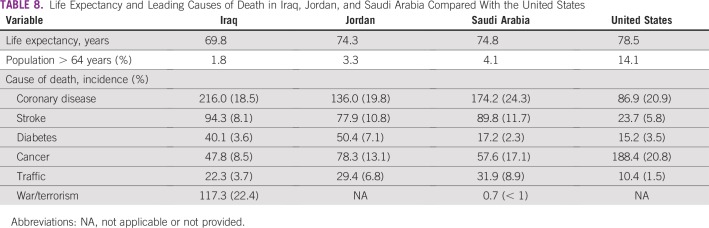
Life Expectancy and Leading Causes of Death in Iraq, Jordan, and Saudi Arabia Compared With the United States

Of factors that may contribute to lymphoma development in the Middle East, *Helicobacter pylori* is found in a large majority of adults, EBV is acquired almost universally in childhood, rates of human herpesvirus-8 and hepatitis B and C are low, and autoimmune disease seems to occur at a frequency similar to American whites (Data Supplement).^30-39^ Only EBV seems to contribute to lymphoma rates, and that to childhood BL. Despite the high prevalence of *H pylori* infection in Iraq, mucosal-associated lymphoid tissue lymphomas are rare and not notably different than in US SEER.^9^

It is estimated that cancer will increase by 70% in the developing world in the next 25 to 30 years.^40^ This is projected because populations worldwide are aging, and cancer is largely a disease of the aged. HL in the Middle East may already be in a transitional phase, with nodular sclerosis exceeding mixed cellularity subtypes and with AAIRs approaching that of the United States. As the Middle East becomes more generally affluent, NHL could follow a transition toward higher incidence rates in older members of the population, such as was seen in US SEER in the 20 years between 1977 and 2013.^9^ Alternatively, current rates could remain low and reflect a genuine variability in NHL oncogenesis.
